# An exploratory investigation of geographic disparities of stroke prevalence in Florida using circular and flexible spatial scan statistics

**DOI:** 10.1371/journal.pone.0218708

**Published:** 2019-08-30

**Authors:** Shamarial Roberson, Rahel Dawit, Jaleesa Moore, Agricola Odoi

**Affiliations:** 1 Bureau of Chronic Disease Prevention, Division of Community Health Promotion, Florida Department of Health, Tallahassee, Florida, United States of America; 2 Biomedical and Diagnostic Sciences, College of Veterinary Medicine, The University of Tennessee, Knoxville, Tennessee, United States of America; University of Zurich, SWITZERLAND

## Abstract

**Background:**

Stroke is a major public health concern due to the morbidity and mortality associated with it. Identifying geographic areas with high stroke prevalence is important for informing public health interventions. Therefore, the objective of this study was to investigate geographic disparities and identify geographic hotspots of stroke prevalence in Florida.

**Materials and methods:**

County-level stroke prevalence data for 2013 were obtained from the Florida Department of Health’s Behavioral Risk Factor Surveillance System (BRFSS). Geographic clusters of stroke prevalence were investigated using the Kulldorff’s circular spatial scan statistics (CSSS) and Tango’s flexible spatial scan statistics (FSSS) under Poisson model assumption. Exact McNemar’s test was used to compare the proportion of cluster counties identified by each of the two methods. Both Cohen’s Kappa and bias adjusted Kappa were computed to assess the level of agreement between CSSS and FSSS methods of cluster detection. Goodness-of-fit of the models were compared using Cluster Information Criterion. Identified clusters and selected stroke risk factors were mapped.

**Results:**

Overall, 3.7% of adults in Florida reported that they had been told by a healthcare professional that they had suffered a stroke. Both CSSS and FSSS methods identified significant high prevalence stroke spatial clusters. However, clusters identified using CSSS tended to be larger than those identified using FSSS. The FSSS had a better fit than the CSSS. Most of the identified clusters are explainable by the prevalence distributions of the known risk factors assessed.

**Conclusions:**

Geographic disparities of stroke risk exists in Florida with some counties having significant hotspots of high stroke prevalence. This information is important in guiding future research and control efforts to address the problem. Kulldorff’s CSSS and Tango’s FSSS are complementary to each other and should be used together to provide a more complete picture of the distributions of spatial clusters of health outcomes.

## Introduction

Stroke occurs when there is an interruption of blood supply to a section of the brain [1]. In the US a stroke event occurs every 40 seconds and someone dies from stroke every 3 minutes 42 seconds [2–4]. The condition is the 5^th^ leading cause of death in Florida and is a significant public health concern because of its associated mortality and long term disability [5,6]. Its economic burden in Florida and the US is estimated at $5.5 billion and $73.7 billion annually, respectively [7,8]. Post-stroke care in the US is reported to be $4,850 per month per patient and is much higher than the cost in Australian ($752 per month per patient) [9].

Geographic disparities of stroke exist in the US with parts of the Southeastern states having very high rates of the condition and are labeled the “stroke belt” [10,11]. For Florida, there is evidence of geographic disparities in stroke hospitalizations and deaths with the highest rates of both being observed in the rural northern parts of the state [12,13]. Unfortunately, not much is known regarding disparities in the prevalence of the condition and yet this information is critical for informing health planning, disease control programs and for shaping health policy to eliminate geographic disparities in stroke prevalence. Moreover, identifying spatial patterns of stroke risk factors is important for identifying populations potentially at high risk of the condition and hence would be useful for targeting resources for health prevention programs [1,14]. Thus, identifying counties with significantly high prevalence of stroke and those with high prevalence of its risk factors will help guide resource allocation and intervention programs [2,15]. This would be instrumental towards meeting one of the goals of the Healthy People 2020 of eliminating health disparities [16]. Healthy People is a program, by the US Department of Health and Human Services, that provides science-based national objectives for improving the health of the nation. It sets benchmarks bench-marks and monitors progress so as to assess the impact of prevention efforts [16] Thus, the objective of this study was to investigate geographic disparities of stroke prevalence and identify its geographic hotspots in Florida using two spatial cluster detection methods.

## Materials and methods

### Ethical statement

This study was approved by the Florida Department of Health as exempt from federal regulations governing research involving human participants: 45 CFR 46.101(b)(4) Research involving the collection or study of existing data, documents, records, pathological specimens, or diagnostic specimens, if these sources are publicly available or the information is recorded by the Investigator in such a manner that subjects cannot be identified, directly or through identifiers linked to the subjects.

### Study design, study area and data source

This ecological study was performed in Florida which had an adult population of more than 15 million people in 2013. Although 70% of Florida land is designated as rural, only approximately 9% of the population lives in rural areas. Stroke prevalence and modifiable risk factor data for 2013 were obtained from the Florida Department of Health’s Behavioral Risk Factor Surveillance System (BRFSS). County level weighted prevalence estimates and 95% confidence intervals for stroke prevalence and its risk factors were computed. Risk factors considered were proportions of county population that reported hypertension, high cholesterol, heavy alcohol consumption, smoking, diabetes, coronary heart disease, overweight or obesity, and physical inactivity.

### Statistical and geographic analyses

All descriptive statistics were performed in SAS [17] while detection of spatial clusters were performed using Kulldorff’s spatial scan statistics (CSSS) and Tango’s spatial scan statistics (FSSS).

#### Kulldorff’s circular spatial scan statistic (CSSS)

Kulldorff’s CSSS, implemented in SaTScan [18], was used to test for the presence of high prevalence stroke spatial clusters and to identify their locations [19,20]. The statistic uses a circular window of variable radius that moves across the study area. The radius of the window varies from 0 to a user-specified maximum. As the window moves across the study area, it defines a set of different neighboring geographical units (counties in this study). If the window contains the centroid of a county, the whole county is included in the window [19]. The approach compares the number of cases within the window with the number expected if cases are randomly distributed in space. Significance of potential clusters is based on a likelihood ratio test whose p-value is obtained through Monte Carlo testing. In this study, purely spatial high-prevalence stroke clusters were investigated under the discrete Poisson probability model assumption using a maximum spatial window size of 13% of the population of the study area. The size of the maximum spatial window was chosen to ensure that all spatial units, including the largest unit (county) which had a population of 13% of the study area population, had a chance to be a cluster and to ensure that the clusters are not unrealistically large as would happen if we used a larger window size. Only non-overlapping clusters were investigated and identified. A total of 999 Monte Carlo replications were performed for statistical inference. The null hypothesis of no clusters was rejected when the simulated p-value was less than or equal to 0.05. Only clusters with prevalence ratio (PR) greater that 1.2 were reported to avoid reporting very low risk clusters.

#### Tango’s flexible spatial scan statistic (FSSS)

Tango’s FSSS approach works in pretty much the same general way as Kulldorff’s CSSS method described above. However, the spatial scanning window in Tango’s FSSS is flexible in shape and not permanently circular. This enables this approach to detect both circular and noncircular clusters. The maximum spatial scanning window size was set at 10 counties specifying Poisson probability model. Restricted log-likelihood ratio (LLR) and 999 Monte Carlo replications were used for statistical inference [21]. The most likely clusters were ordered based on their restricted LLR and the cluster with the largest value was identified as the primary cluster. As for Kulldorff’s CSSS, the null hypothesis of no clusters was rejected when the simulated p-value was less than or equal to 0.05. Finally, as was done for the CSSS, only clusters with prevalence ratio (PR) greater that 1.2 were reported to avoid reporting very low risk clusters.

#### Comparison of results of Kulldorff’s CSSS and Tango’s FSSS approaches

Two-sample test of equality of proportions with continuity correction, implemented in R [22], was used to compare the proportion of cases and population living in the cluster counties identified by the two methods. Exact McNemar’s test, computed in R [22] using the stats package [22], was used to compare the proportion of counties identified as belonging to a cluster by each of the two methods. Additionally, Cohen’s Kappa statistics as well as prevalence and bias adjusted Kappa (PABAK or *S* coefficient) [23,24] were computed in R [22], using the epiR package [25], to assess the level of agreement between Kulldorff’s CSSS and Tango’s FSSS methods of cluster detection. Interpretation of Kappa results were done using the categories proposed by Landis and Koch [26]. Briefly, interpretation of Kappa results following the above method is as follows:

Kappa values <0 indicates "No agreement", values 0–0.2 indicate "Slight agreement", 0.2–0.4 imply "Fair agreement", 0.4–0.6 imply "Moderate agreement", 0.6–0.8 imply "Substantial agreement" while values 0.8–1.0 indicate "Almost perfect agreement". Finally, goodness of fit of the models were compared using Cluster Information Criterion (CLIC) computed as follows:

CLIC=-2*ΣLLRs+log(p)*n

Where, ΣLLRs is the sum of the log likelihood ratios, p is the population included in the identified clusters and n is the number of significant clusters.

#### Adjusting cluster detection for known risk factors (covariates)

A limitation of cluster investigation without adjusting for known risk factors is that it is unclear which risk factors are important in explaining the identified spatial clusters. Therefore, it is important to adjust for known risk factors of the outcome and especially if the known risk factors are not randomly distributed in space. Doing this helps to identify clusters that are not explained by the known risk factors adjusted for. Thus, to assess which of the identified clusters were wholly or partially explained by the known risk factors of stroke and which were not explained by the risk factors, a CSSS analysis needs to be performed after adjusting for the known risk factors. The known risk factors of stroke considered for adjustment were: % of population in each county that self-reported angina, physical inactivity, overweight/obese, hypertension, high cholesterol, diabetes and smoking. Since SaTScan cannot adjust for continuous covariates, the covariate adjustment was done in two steps. In the 1^st^ step a Poisson model was fit to the data in STATA with the number of cases of stroke in each county specified as the outcome and the known risk factors listed above as explanatory variables while the county population was specified as the offset. Based on this model, the expected number of cases was computed for use in the 2^nd^ step. In the 2^nd^ step the computed covariate adjusted expected number of cases (from step 1) for each county was used to replace the raw population numbers in the CSSS model for investigating the clusters. The rest of the model specification remained as described in the Kulldorff’s CSSS section.

### Mapping

All cartographic manipulations and displays were performed in ArcGIS 10.5 [27]. The choropleth maps of stroke prevalence as well as prevalence of investigated risk factors were generated using Jenk’s optimization classification scheme to determine the critical intervals for mapping. Identified high prevalence stroke spatial clusters were also displayed using ArcGIS 10.5 [27].

## Results

### Stroke prevalence

The overall prevalence of stroke was 3.7% and was the same among men and women (Table 1). Seniors (≥65 years old) had the highest prevalence (7.8%) while the lowest prevalence was observed among the 18–44 year olds (1.1%) (Table 1).

**Table 1:** Overall and factor-specific prevalence of stroke among adults ≥ 18 years in Florida, 2013.

Factors		Stroke Prevalence (%)	95% CI Lower Bound	95% CI Upper Bound
**Overall**		3.7	3.3	4.1
**Sex**				
	Male	3.7	3.1	4.2
	Female	3.7	3.2	4.2
**Age Group**				
	18–44	1.1	0.7	1.6
	45–64	4.1	3.4	4.8
	65 & Older	7.8	6.9	8.7
**Race/Ethnicity**				
	Non-Hispanic White	4.3	3.8	4.7
	Non-Hispanic Black	4.6	3.1	6.1
	Hispanic	2.0	1.2	2.7
**Education**				
	<High School	5.2	3.9	6.5
	High School/GED	3.7	3.0	4.3
	>High School	3.3	2.9	3.8
**Annual Income**				
	<$25,000	5.7	4.8	6.5
	$25,000-$49,999	4.1	3.3	4.9
	$50,000 or More	1.9	1.4	2.3
**Marital Status**				
	Married/Couple	3.3	2.8	3.8
	Not Married/Couple	4.2	3.6	4.7

Based on race and ethnicity, the prevalence was highest among Non-Hispanic Blacks (4.6%) and lowest among Hispanics (2%). Interestingly, the prevalence of stroke among Non-Hispanic Whites (4.3%) was not significantly different from Non-Hispanic Blacks (4.6%), however both were significantly higher than the prevalence among Hispanics (Table 1). Stroke prevalence was highest (5.2%) among adults that had less than high school education and lowest (3.3%) among those with higher than high school education (Table 1). Moreover, individuals with lowest annual income had the highest prevalence (5.7%) while those with highest annual income had the lowest prevalence (1.9%). Stroke prevalence was also higher among the non-married individuals (4.2%) than those who were married (3.3%).

### Geographic distribution of stroke and stroke risk factors

**Fig 1:**
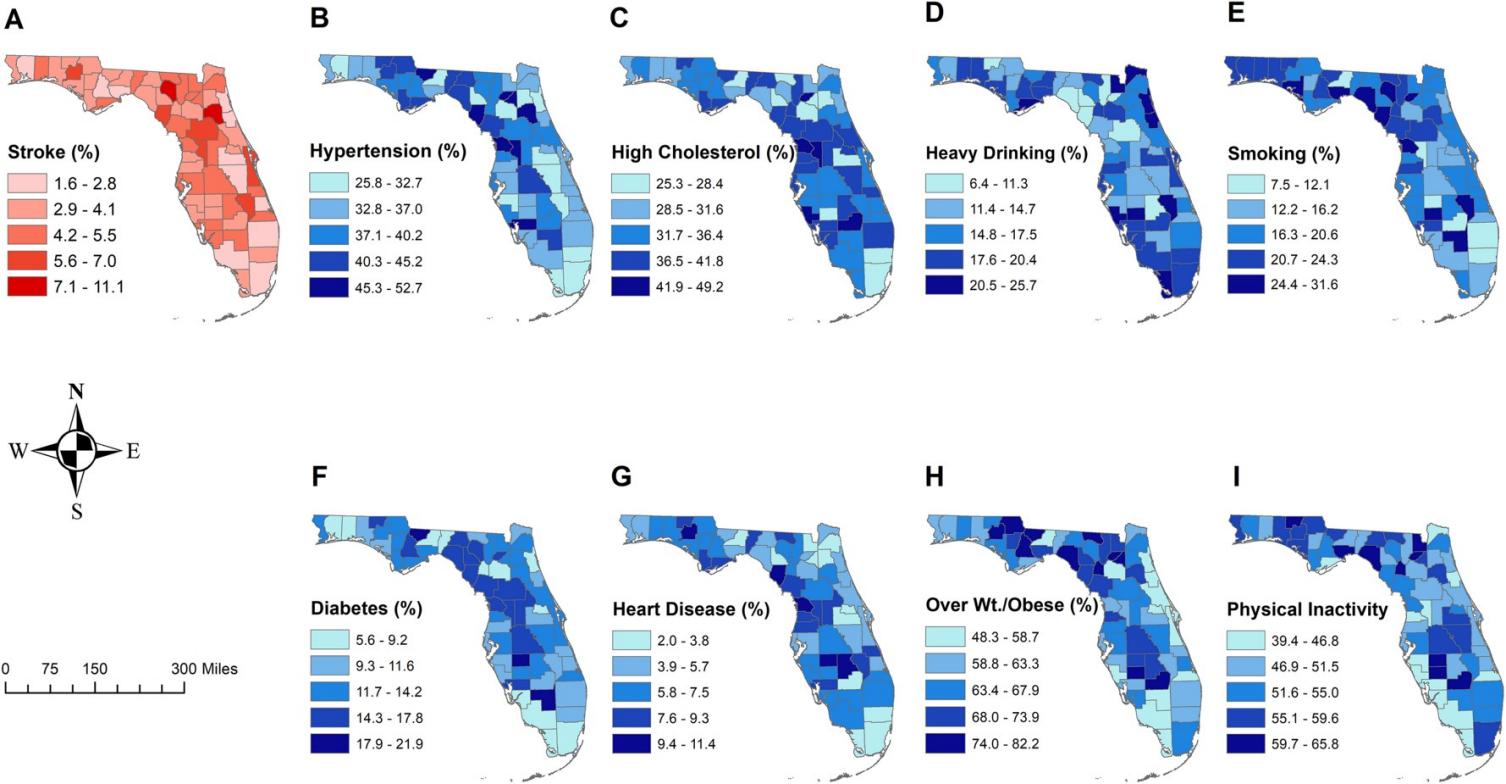
Geographic distribution of stroke prevalence and prevalence of some of its risk factors in Florida, 2013 (Reprinted with permission from Esri, ArcMap, Florida Department of Health, and the GIS User Community under a CC-BY license, original copyright 2018).

The geographic patterns of stroke prevalence varied by geographical region ranging from 1.6% to 11.1%, with higher prevalence proportions being observed in the northcentral and central part of the state and lower prevalence being observed in the south and some urban counties in the north (Fig 1). The northcentral and central parts of the state, which had higher prevalence proportions, are generally more rural than the south.

**Fig 2:**
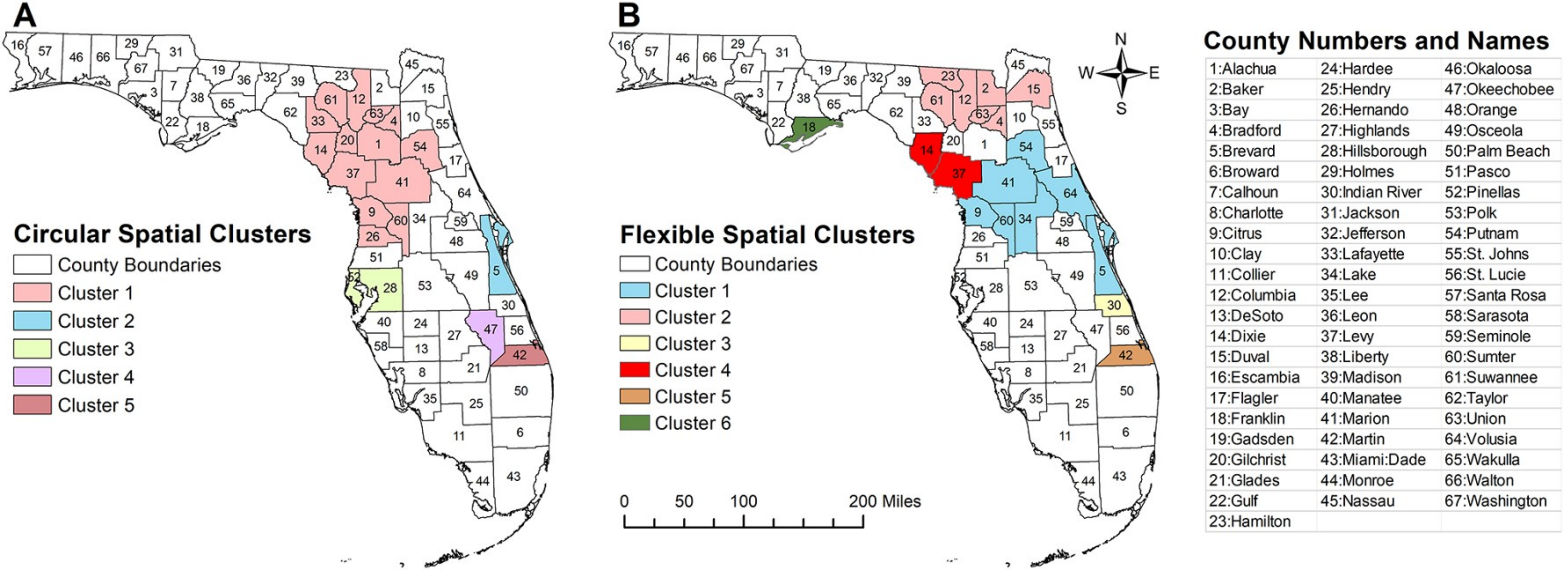
Geographic distribution of high prevalence stroke spatial clusters with prevalence ratios > 1.2 that were identified in Florida using Kulldorff’s circular spatial scan statistics and Tango’s flexible spatial scan statistics, 2013. (Reprinted with permission from Esri, ArcMap, Florida Department of Health, and the GIS User Community under a CC-BY license, original copyright 2018).

Geographic distribution of stroke risk factors was very similar to those of stroke prevalence (Fig 1). The overall prevalence of diabetes was 11.2% (Table 2) but varied from 1.6% to 11.1% (Fig 1). Moreover, the prevalence of diabetes showed similar geographic patterns as stroke prevalence, with higher prevalence being observed in the northcentral and central counties. Similarly, high prevalence of coronary heart disease was observed in central counties while lower prevalence proportions were observed in the northeastern and southern counties. Hypertension prevalence was highest in a rural county (Dixie, 52.7%) and the lowest in an urban county (Leon, 25.8%), (Figs 1 and 2). Counties with high prevalence of hypertension tended to be in the northwest, northcentral and central parts of the state. Similar spatial patterns were observed for cholesterol prevalence (Fig 1).

**Table 2:** Prevalence of stroke risk factors among adults ≥ 18 years, by selected characteristics in Florida, 2013.

Factors		Prevalence (%)	95% CI^1^ Lower Bound	95% CI^1^ Upper Bound
Physical Activity				
	Physically Inactive	52.9	51.6	54.3
	Active	47.1	45.7	48.4
Body Mass Index				
	Under/Normal Weight	37.2	36.0	38.4
	Overweight/ Obese	62.8	61.6	64.0
Hypertension				
	Hypertension	34.6	33.5	35.7
	Normal/Borderline	65.4	64.3	66.5
Angina/CHD		5.0	4.6	5.4
Cholesterol Level				
	High	40.3	39.1	41.6
	Normal	59.7	58.4	60.9
Diabetes		11.2	10.5	11.9
Smoking Status				
	Current Smoker	16.8	15.9	17.7
	Former Smoker	28.1	27.1	29.2
	Never Smoke	55.0	53.8	56.2
Drinking Status	Heavy/Binge Drinking	17.6	16.6	18.6

^1^Confidence Interval

The overall prevalence of heavy alcohol consumption or binge drinking was 17.6% (Table 2). Interestingly, high prevalence of alcohol consumption was observed throughout the state except in a few counties in the central part of the state. Monroe County had the highest prevalence of heavy or binge drinking (25.7%) while Union County had the lowest (6.4%) (Fig 1). As for smoking, the Florida panhandle counties located in northwestern and northcentral part of the state had higher prevalence, while the southeastern part of the state had lower prevalence than the state average (Fig 1). The overall Florida prevalence of overweight or obesity was 62.8% with the highest prevalence proportion being reported in Liberty County (82.2%) and the lowest in Martin County (48.3%) (Figs 1 and 2). Counties in the northcentral and central parts of the state again had higher prevalence while, the east-central and southeastern part of the state had a lower prevalence than the rest of the state. Finally, the overall prevalence of physical inactivity was 52.9% (Table 2) with the highest levels being observed in the northwest, northcentral and central parts of the state while the lowest prevalence proportions were observed in southwestern part of the state.

### Clusters of high stroke prevalence (comparison of results of Kulldorff’s CSSS and Tango’s FSSS)

Table 3 and Fig 2A show the characteristics and spatial distribution of significantly high prevalence geographic clusters/hotspots, identified using Kulldorff’s CSSS and that had prevalence ratio (PR) > 1.2. This approach identified 5 clusters that comprised a total of 19 counties. The primary cluster had the largest number of counties and a PR of 1.61, implying that the prevalence of stroke in this cluster was 61% higher than the state average. These counties were located in the northcentral part of the state (Fig 2A). All secondary clusters were composed of one county each, except cluster 3 which had 2 counties. Cluster 4 had the highest prevalence ratio (PR = 1.91) while cluster 5 had the lowest (PR = 1.23).

**Table 3:** High prevalence stroke spatial clusters in Florida identified using Kulldorff’s circular spatial scan statistics and Tango’s flexible spatial scan statistics.

Cluster	Population	Observed # of cases	Expected # of cases	Prevalence Ratio	# of counties in cluster	P-value
	**Kulldorff’s Circular Spatial Scan Statistics Identified Clusters**					
1	1,314,154	74,397	48,017.14	1.61	14	<0.001
2	549,144	32,949	20,064.86	1.67	1	<0.001
3	2,210,618	97,622	80,772.54	1.24	2	<0.001
4	39,770	2,784	1,453.12	1.91	1	<0.001
5	148,189	6,669	5,414.59	1.23	1	<0.001
	**Tango’s Flexible Spatial Scan Statistics Identified Clusters**					
1	2,009,003	112,106	73,405.80	1.53	7	0.001
2	1,074,486	50,139	39,260.00	1.28	7	0.001
3	139,787	7,129	5,107.60	1.40	1	0.001
4	56,629	3,212	2,069.14	1.55	2	0.001
5	148,189	6,669	5,414.60	1.23	1	0.001
6	11,613	592	424.32	1.40	1	0.001

The distribution and characteristics of high prevalence clusters, identified using the Tango’s FSSS and that had PR>1.2 is shown in Table 3 and Fig 2B. Although this approach identified 6 clusters (1 cluster more than Kulldorff’s CSSS method), it also identified a total of 19 counties as being parts of high prevalence stroke clusters. Additionally, the clusters identified using Tango’s FSSS were generally smaller than those identified by Kulldorff’s CSSS. Moreover, those involving more than one county were irregularly shaped unlike those identified by Kulldorff’s CSSS that tended to be circular (Fig 2A and 2B). The primary FSSS cluster had 7 counties and a PR of 1.53 implying that the prevalence of stroke in this cluster was 53% higher than the Florida average. The lowest prevalence cluster identified by Tango’s FSSS also had a PR of 1.23 (Table 3).

It is worth stressing that the clusters identified by Kulldorff’s CSSS method were generally larger as evidenced by both the large geographic extent of the primary cluster identified by CSSS method as well as the larger number of cases and population involved in these clusters. For instance, the total number of cases involved in the clusters identified by Kulldorff’s CSSS method (214,421) was much higher than those involved in the clusters identified by Tango’s FSSS method (179,847). Thus, the proportion of cases included in clusters identified by Kulldorff’s CSSS method (30.4%; 214,421/705,718) were significantly (p<0.0001) higher than that identified by Tango’s FSSS (25.5%; 179,847/705,718). Similarly, the total population living in counties identified by Kulldorff’s method as high prevalence counties was also much higher (approx. 4.3 million) compared to the population living in counties identified by Tango’s FSSS method (3.4 million) (Table 4). Comparison of the proportions again revealed that the proportion of the population in cluster counties identified by Kulldorff’s CSSS were significantly (p<0.0001) higher (22.1%; 4,261,875/19,314,396) than that of counties identified as parts of a cluster by Tango’s FSSS method (17.8%; 3,439,707/19,314,396).

**Table 4:** Comparison of characteristics of the results of Kulldorff’s circular spatial scan statistics and Tango’s flexible spatial scan statistics.

Item	Kulldorff’s circular spatial scan statistics	Tango’s flexible spatial scan statistics
Number of counties in primary and largest cluster	14	7 (2 clusters had 7 counties)
Total number of counties identified belonging to a high-prevalence cluster	19	19
Number of counties identified as belonging to a high- prevalence cluster by both methods	12	12
List of counties identified as belonging to a high- prevalence cluster by both methods	Bradford, Brevard, Citrus, Columbia, Dixie, Levy, Marion, Martin, Putnam, Sumter, Suwannee, and Union.	Bradford, Brevard, Citrus, Columbia, Dixie, Levy, Marion, Martin, Putnam, Sumter, Suwannee, and Union.
Number of counties identified as belonging to a high- prevalence cluster by only one of the methods	7	7
List of counties identified as belonging to a high-prevalence cluster by only one of the methods	Alachua, Gilchrist, Hernando, Lafayette, Hillsborough, Pinellas, Okeechobee	Baker, Duval, Franklin, Hamilton, Indian River, Lake, Volusia.
Total number of counties classified as non-cluster counties	41	41
Total number of cases in high-prevalence cluster counties	214,421	179,847
Total population in high-prevalence cluster counties	4,261,875	3,439,707
Cluster Information Criterion (CLIC)	25,532	23,814

It is interesting to note that both methods identified the same proportion of cluster positive counties (28%; 19/67). Thus, as would be expected, use of exact McNemar’s test to compare the proportion of counties identified as belonging to a cluster by the two methods indicated no evidence that the two proportions differed (p = 1.0). However, although both methods identified 19 counties as belonging to a high prevalence cluster and 41 counties as not being part of a cluster, the counties identified by the methods as being part of a cluster were not identical. Both methods agreed in the identity of only 12 of the 19 counties identified by both methods as being part of a cluster implying that they each identified 7 additional counties, not identified by the other method, as belonging to a cluster (Tables 4 and 5).

**Table 5:** Contingency table showing the distribution of counties across clusters identified by Kulldorff’s CSSS and Tango’s FSSS cluster detection methods.

		**Kulldorff’s CSSS**^1^		
		**Cluster +**	**Cluster -**	**Total**
**Tango’s FSSS**^2^	**Cluster +**	12	7	19
	**Cluster -**	7	41	48
	**Total**	19	48	67

^1^Circular Spatial Scan Statistics

^2^Flexible Spatial Scan Statistics

The observed proportion of agreement between the two methods was 79.1% while the expected proportion of agreement by chance alone was 59.37%. Thus, the two methods had moderate agreement with each other (Cohen’s Kappa = 0.4857 (95% Confidence interval (CI): 0.2462, 0.7253; p<0.0005). It is worth noting that the prevalence and bias adjusted Kappa (PABAK or *S* coefficient), estimated at 0.5821 (95% CI: 0.3487, 0.7616; p<0.0001), resulted in the same conclusion (moderate agreement between the two methods). Finally, assessment of goodness of fit of the models showed that Tango’s FSSS had a better fit (CLIC = 23,814) than Kulldorff’s CSSS (CLIC = 25,532) since the cluster information criterion (CLIC) of Tango’s FSSS was lower than that of Kulldorff’s CSSS (Table 4).

### Covariate adjusted spatial clusters

**Fig 3:**
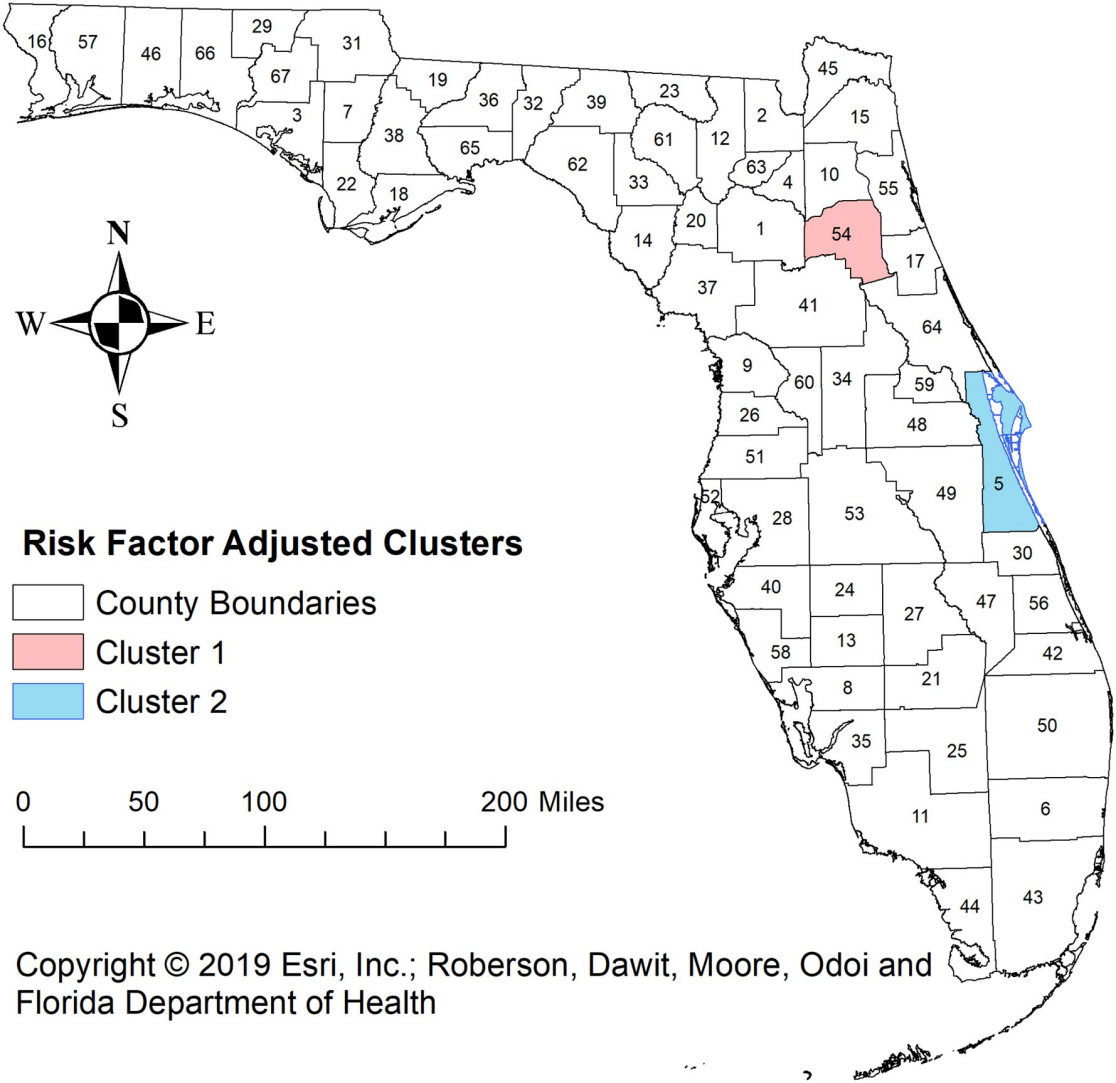
Geographic distribution of risk factor adjusted high prevalence stroke spatial clusters with prevalence ratios > 1.2 that were identified in Florida using Kulldorff’s circular spatial scan.

Table 6 and Fig 3 show the characteristics and spatial distribution of covariate (risk factor) adjusted significantly high prevalence spatial clusters with PR>1.2 identified using Kulldorff’s CSSS. Only one of the full clusters and part of another cluster originally identified by unadjusted Kulldorff’s CSSS were identified in this covariate adjusted analysis. This indicates that the high prevalence observed among the other three clusters that were identified by the unadjusted analyses but not by the adjusted analysis were fully explained by the risk factors adjusted for in the Poisson model. The primary cluster identified by the adjusted analysis had a PR of 2.53 and was comprised of only one county (Putman County) which was one of the 14 counties included in the primary cluster of the unadjusted analysis. This implies that although the risk factors included in the model explained the high stroke prevalence observed in the other 13 counties that formed cluster 1 of the unadjusted analysis, they did not explain the high prevalence observed in Putman county. The 2^nd^ cluster identified by the covariate adjusted CSSS was also cluster 2 identified by the unadjusted analysis and comprised only one county (Brevard County). In the adjusted analysis, this cluster had a PR = 1.25 but it had a PR = 1.67 in the unadjusted analysis.

**Table 6:** Covariate (risk factor) adjusted high prevalence stroke spatial clusters in Florida identified using Kulldorff’s circular spatial scan statistics.

Cluster	Observed # of cases	Expected # of cases	Prevalence ratio	# of counties in cluster	P-value
1	8,052	3,207.44	2.53	1	<0.001
2	32,949	26,527.38	1.25	1	<0.001

## Discussion

The objective of this study was to investigate geographic disparities and identify high prevalence hotspots of stroke in Florida. Florida is a very diverse state both geographically and population heterogeneity. Therefore, examining the geographic disparities in the burden of stroke and related risk factors is important for public health planning and intervention. The use of spatial statistical epidemiologic approaches, such as those used in this study, enhance detection of significant hotspots of disease and is critical for guiding evidence-based intervention and prevention efforts to reduce disparities and improve population health for all Floridians.

### Comparison of Kulldorff’s CSSS and Tango’s FSSS

There is evidence of significant high stroke prevalence geographic hotspots based on both the CSSS and FSSS results. Although Kulldorff’s CSSS has been used in a variety of epidemiological investigations of event clusters, it uses a circular window to define the potential cluster areas and therefore does not do a very good job of correctly detecting actual non-circular clusters [28]. Unfortunately it is reported that, compared to CSSS, the cluster detection method proposed by Duczmal and Assunção [29] for detection of noncircular clusters tends to detect clusters that are much larger than their true size [28]. Studies have been performed to compare the performance of Kulldorff’s CSSS and Tango’s FSSS methods used in this study. For instance, Tango and Takahashi have shown that Kulldorff’s CSSS has a high level of accuracy in detecting circular clusters. While Tango’s FSSS had good power but not quite as high as that of Kulldorff’s CSSS, it had the additional strength of detecting noncircular high-risk clusters more accurately than the Kulldorff’s CSSS [28]. Moreover, they showed that Kulldorff’s CSSS had 0 power for detecting noncircular clusters that cannot be detected by circular windows. Kulldorff’s CSSS also has a tendency of identifying larger clusters than their true sizes even when the true shape of the cluster is circular. The cost of these large clusters, they discussed, is the larger population misclassified as belonging to a cluster [28]. In fact, this is consistent to the findings of our study where we found that the population classified as belonging to a cluster by Kulldorff’s CSSS was much larger (4.3 million) than that of Tango’s FSSS (3.4 million), a 24% difference. They (Tango and Takahashi) concluded that the FSSS worked well for small to moderate cluster sizes of no more than 30 areas and is not feasible for larger clusters [28]. A strength of Tango’s FSSS over Kulldorff’s CSSS is the option to use restricted log likelihood ratio that only scans areas of elevated risk and therefore ensures that no areas of low risk are included as potential clusters. Use of restricted log likelihood ratio has been shown to result in better ability to identify true clusters compared to Kulldorff’s CSSS [30]. At the moment, Kulldorff’s CSSS implementation in SaTScan [18] is not able to do this and hence invariably includes some low risk areas as part of a disease cluster.

In this study, our findings revealed that the two methods had moderate agreement in identifying clusters as their observed agreement was 79.1% with a bias adjusted kappa of 0.5821. However, the overall goodness-of-fit test indicated that Tango’s FSSS had a better fit. A study by Goranson et al (2008) reported that noncircular clusters with high relative risk were detected by Tango’s FSSS but not Kulldorff’s CSSS [31]. Similar to our findings, they also reported that the p-values of the cluster identified by Kulldorff’s CSSS method tended to be smaller than those of clusters identified by Tango’s FSSS. They concluded that the two methods are complementary to each other and should be used together because while the CSSS was more useful for identification of more circular clusters, the FSSS was better at identifying noncircular clusters [31]. Similar recommendations have been made by Tango (2008) [30]. Therefore, used together, they may provide the best clues to understanding disease distributions and in detection of disease outbreaks.

### Distribution of stroke clusters

Most of the high stroke prevalence hotspots were in the northcentral and central parts of Florida. The observed geographic disparities are also consistent with findings from the REGARDS study which reported presence of geographic disparities of stroke mortality as a result of disparities in stroke incidence and case fatality rates [32]. A number of other studies have also reported geographic disparities and spatial clusters of stroke [12,33–35]. The observed high prevalence clusters in northcentral Florida are consistent with reports by Siegel et al [8], who suggested that north Florida is part of the stroke belt. The fact that past investigations identified stroke clusters only in the north and not south or central parts of the state and yet this study identified clusters both in the north and central counties may suggest that the stroke belt might be advancing further south. This calls for regular assessment of these spatial patterns to assess changes over time and to guide prevention programs regarding targeted allocation of resources aimed at reducing disparities, stroke risk factors and stroke prevalence.

Contrary to our findings, some studies have not found geographic overlap between stroke clusters and distribution of its risk factors [33,36]. However, the geographic disparities in stroke risk factors observed in the current study is important as it may help explain the disparities in stroke prevalence [37] and may be indicative of counties that are likely to have high prevalence in the near future (a factor of current prevalence of risk factors). In other words, counties that may not currently be part of a stroke cluster but have high prevalence proportions of hypertension, high cholesterol, high alcohol consumption, diabetes, coronary heart disease, obesity and physically inactivity (which are known risk factors for stroke) are highly likely to become stroke clusters in future unless something is done to address these problems. Thus, the findings of this study regarding the geographic distribution of the risk factors provided in this study is critical as it provides useful information to guide health planning, prevention and health promotion programs. Thus, the findings from this study will provide information to guide evidence-based targeting of resources to reduce the prevalence of stroke risk factors and hence prevent stroke, reduce health disparities and improve the health of the entire population of Florida. Therefore, it may be useful in targeting preventive efforts.

With regard to hypertension, the results of this study were consistent with those of other studies that reported higher hypertension prevalence in rural than urban areas [38–40]. A study conducted in Turkey reported higher prevalence of hypertension in rural areas and suggested that it might be due to the migration of younger individuals out of rural areas, which results in older population residing in rural areas [41]. This might be the case in our study as well. Furthermore, Baernholdt et al. reported higher percentage of older adults living in rural counties in the US [42] and may, in part, explain the higher burden of stroke in rural areas seen in our study [43]. Moreover, stroke prevalence tends to be higher in older populations and hence counties with higher percentage of older adults tend to have higher stroke prevalence. Thus, intervention programs should strategically target the rural counties that have more older adults.

Stroke is also associated with sedentary lifestyle, obesity and nutrition. In the current study, coronary heart disease, diabetes and cholesterol prevalence had similar geographic patterns as stroke prevalence. A Chinese study reported that regions with high prevalence of stroke were accompanied with high prevalence of high cholesterol, hypertension and lack of physical activity [41]. In general, counties with higher prevalence of stroke risk factors in this study tended to have higher prevalence of stroke. Unfortunately, further investigation to statistically assess the association between stroke prevalence and its risk factors was beyond the scope of this study whose aim was to investigate stroke disparities and identify geographic hotspots. However, a follow-up study will use global and local models to further investigate the statistical associations between stroke disparities and its risk factors as well as identify the most important factors in different locales across the state.

It is interesting and encouraging to note that most of the unadjusted CSSS clusters disappeared after adjustment for known risk factors assessed in this study. This implies that these risk factors explain the occurrence of most of the identified clusters with the exception of two clusters: the one county cluster in Brevard County and only 1 of the 14 counties that was part of the primary unadjusted cluster. Since stroke has several risk factors, these two identified adjusted clusters may be due to risk factors not adjusted for in this investigation. Suffice it to say that these findings are useful in guiding resource allocation and intervention programs [2,15]. Such programs could focus on reducing the prevalence of the assessed risk factors with special attention given to the areas which were clusters in the unadjusted analysis but not part of a cluster in the adjusted analysis. However, since this is an exploratory study, more detailed investigations will be needed to further investigate these relationships and their geographic disparities to better guide control efforts.

The strength of both the CSSS and FSSS is that they both adjust for multiple testing by only specifying the maximum possible cluster size. A limitation of this study is that estimates of stroke prevalence are based on self-reports and hence may be under-estimated and should be interpreted with that knowledge in mind. Another limitation of the study is maximum scanning window size selected for the analysis of CSSS and FSSS. The cluster sizes that are produced may vary depending on the window size selected for the analysis. In this study, our maximum spatial window size was based on biological considerations and knowledge of the geography of the study area.

## Conclusion

There is evidence of geographic disparities of stroke prevalence with hotspots identified in the northcentral and central parts of the state. Most of these clusters disappeared after adjusting for known risk factors implying that the assessed risk factors may be determinants of occurrence of these clusters. Although the results of CSSS and FSSS are similar, the latter has a better fit and is better for identifying noncircular clusters. However, the two methods complement each other and should be used together so as to get the best picture of the geographic distribution of disease clusters. Using spatial analysis to investigate chronic disease burden and identify high risk communities is useful in guiding strategic planning initiatives aimed at reducing and eliminating disparities. Future studies will investigate determinants of identified hotspots and approaches needed to reduce geographic disparities. Finally, the results of this study will be used to drive a call-to-action for stroke prevention efforts in Florida. The Florida Department of Health is working with various community partners, local county health departments and city governments to implement programs that increase awareness of stroke and its risk factors.
